# Supplementary material for: Neurovascular Coupling Impairment in Heart Failure with Reduction Ejection Fraction
Source: Brain Sci. 2020 Oct 7;10(10):714. doi: 10.3390/brainsci10100714 (PMC7601077; doi:10.3390/brainsci10100714)
Supplement: Supplementary file 1 [file brainsci-10-00714-s001.pdf]

Supplementary Materials: Neurovascular Coupling Impairment in Heart Failure with Reduction Ejection Fraction

Ana Aires, António Andrade, Elsa Azevedo, Filipa Gomes, José Paulo Araújo and Pedro Castro

Table S1. HF etiology effect on neurovascular coupling (NVC) in heart failure (HF) patients.

Neurovascular Coupling (PCA)	Idiopathic (n = 11)	Alcoholic (n = 8)	Ischemic (n = 8)	p Values *
Overshoot systolic CBFV (%)	20.28 (8.76)	20.19 (6.58)	16.16 (4.21)	0.40
Gain (%)	13.86 (7.14)	14.56 (7.20)	10.63 (6.01)	0.47
Natural frequency (Hz)	0.18 (0.07)	0.21 (0.09)	0.15 (0.03)	0.25
Attenuation (a.u)	0.39 (0.24)	0.41 (0.32)	0.28 (0.24)	0.56
Rate time (s)	1.79 (3.78)	1.48 (3.50)	0.96 (2.54)	0.87

All values are given in mean ± SD. a.u., arbitrary units; CBFV, cerebral blood flow velocity; Hz, Hertz; PCA, posterior cerebral artery; s seconds. * p value of ANOVA for the comparison of means of each group (HFrEF, HFrecEF and heathy controls).
